# Evaluating the factors affecting clinical outcomes in critically ill COVID-19 unvaccinated patients admitted to the intensive care unit in a lower-middle-income country

**DOI:** 10.1097/MS9.0000000000001379

**Published:** 2023-11-20

**Authors:** Fatemeh Heydari, Elahe Karimpour-razkenari, Parnian Azadtarigheh, Alireza Vahdatinia, Ali Salahshoor, Abbas Alipour, Mahmood Moosazadeh, Afshin Gholipour Baradari, Mahila Monajati, Fahimeh Naderi-Behdani

**Affiliations:** a Department of Anesthesiology and Critical Care Medicine, Imam Khomeini Hospital; b Department of Clinical Pharmacy, Faculty of Pharmacy; c Faculty of Medicine; d Community Medicine Department, Medical Faculty; e Gastrointestitional Cancer Research Center, Non-communicable Disease Institute, Mazandaran University of Medical Sciences, Sari; f Ramsar Campus; g Department of Clinical Pharmacy, Faculty of Pharmacy, Ramsar Campus, Mazandaran University of Medical Sciences, Ramsar, Mazandaran Province; h Department of Internal Medicine, Golestan University of Medical Sciences, Gorgan, Iran

**Keywords:** Coronavirus Disease 2019, ICU, Mortality Risk, APACHE II, Lower-Middle-Income, SARS-CoV-2

## Abstract

**Background:**

COVID-19, the most destructive pandemic of this century, caused the highest mortality rate among ICU patients. The evaluation of these patients is insufficient in lower-middle-income countries with limited resources during pandemics. As a result, our primary goal was to examine the characteristics of patients at baseline as well as their survival outcomes, and propose mortality predictors for identifying and managing the most vulnerable patients more effectively and quickly.

**Methods:**

A prospective analysis of COVID-19 ICU-admitted patients was conducted in our healthcare centre in Iran, from 1 April until 20 May 2020. Ninety-three patients were included in the study, and all were unvaccinated. A multi-variate logistic regression was conducted to evaluate mortality-associated factors.

**Results:**

There were 53 non-survivors among our ICU-admitted patients. The mean duration from symptoms’ onset to hospitalization was 6.92 ± 4.27 days, and from hospitalization to ICU admission was 2.52 ± 3.61 days. The average hospital stay for patients was 13.23 ± 10.43 days, with 8.84 ± 7.53 days in the ICU. Non-survivors were significantly older, had significantly lower haemoglobin levels and higher creatine phosphokinase levels compared to survivors. They had marginally lower SpO2 levels at admission, higher vasopressor administrations, and were intubated more significantly during their ICU stay. The use of immunosuppressive drugs was also significantly higher in non-survivors. Logistic regression revealed that a one-point increase in APACHE II score at ICU admission increased mortality by 6%, and the presence of underlying diseases increased mortality by 4.27 times.

**Conclusion:**

The authors presented clinical mortality prediction factors for critically ill patients infected with COVID-19. Additional studies are necessary to identify more generalized mortality indicators for these patients in lower-middle-income countries.

## Graphical abstract

**Figure:**
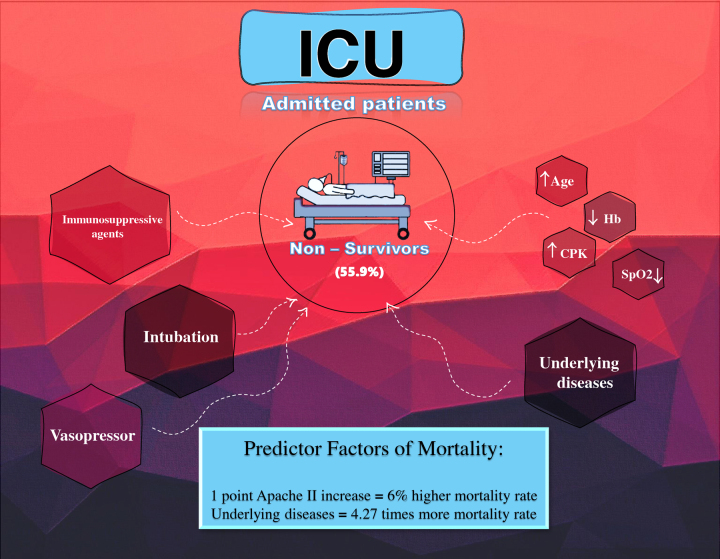

## Introduction

Highlights

- Mortality among COVID-19 ICU-admitted patients is high in lower-middle-income countries.
- The overall mortality rate of our ICU patients was 55.9% at the beginning of the COVID-19 pandemic.
- A one-point increase in APACHE II score at ICU admission increases mortality by 6%.
- The presence of underlying diseases increases mortality by 4.27 times.
- Other factors related to mortality are old age, low haemoglobin levels, increased creatine phosphokinase levels, low levels of oxygen saturation, the use of immunosuppressive agents, the administration of vasopressors, and intubation.

COVID-19 has caused tragedy worldwide, claiming the lives of more than 375 000 people as of 20 May 2020. It stands as the most lethal global health crisis in the past century^1,2^. The rapid spread of the disease globally prompted the WHO to designate it as a pandemic on 11 March 2020^3^. ICU have been a necessity in treating the most severely ill patients infected with this disease^4^. However, the shortage of supplies and equipment required for critically ill ICU patients has placed a heavy burden on the already strained healthcare system^5^.

The mortality rate of patients infected with COVID-19 admitted to the ICU can be differentiated by their geographical area, ethnicity, and even per capita gross national income (GNI), which varied from 42.2 to 98.8% at the beginning of the pandemic^4^. Previous studies have already suggested a number of predictors at baseline for determining the prognosis of COVID-19 patients^6^. Moreover, another factor called the APACHE II score (Acute Physiologic and Chronic Health Evaluation II) has been used to estimate survival rates in critically ill non-COVID-19 ICU-admitted patients^7^.

However, a great number of deaths, especially in lower-middle-income countries, due to this pandemic, and also a small number of studies, mainly about critically ill ICU patients with COVID-19 in these countries, indicate that more reliable data about predictive tools of mortality is needed to provide better care for patients with a higher mortality risk in cases of a shortage of resources. In this study, we analyzed unvaccinated, COVID-19-infected patients who were admitted to the ICU of our medical centre during the pandemic’s beginning in Iran. We evaluated their clinical characteristics at baseline and their survival outcomes. We also suggested significant mortality predictors for these patients at their ICU admission.

## Methods

We conducted this prospective, longitudinal study at our medical and educational hospital, which is a 300-bed referral hospital, designated for severely or critically ill patients with COVID-19. The research was approved by the relevant ethics committee (approval code 1399.351). Patients’ informed written consent (or from legally authorized substitute decision-makers) were obtained. This study meets STROCSS criteria^8^.

### Patients and duration of the study

We enroled adult patients who were admitted to the ICU or transferred from other hospitals, fever clinics, and other hospital sites, with a confirmed diagnosis of COVID-19 with positive reverse transcription-polymerase chain reaction (RT-PCR), from 1 April to 20 May 2020.

### ICU admission criteria

Based on our COVID-19 national protocol^9^, we admitted these patients to the ICU: (1) severe COVID-19 patients with peripheral oxygen saturation (SpO2) lower than 85% and/or severe respiratory distress, such as nasal flaring, air hungry, intercostal retraction, subcostal retraction, and/or hemodynamic instability and/or acid-base disturbance, and (2) moderate to severe patients (SpO2, 85% to 90%, mild to moderate respiratory distress, ill or toxic general appearance) with risk factors such as BMI higher than 30 kg/m^2^, underlying disease, older than 70 years, and immunosuppressive agents using history. Patients not meeting these criteria remained with an intensive monitoring protocol on the ward.

### Outcomes

Our primary outcomes were: (1) to report common characteristics and laboratory data at baseline in our patients and (2) to find mortality predictors for them.

Our secondary outcomes were: (1) to identify the mortality rate and survival time in these patients as well as their length of stay in the hospital and the ICU; (2) to determine the incidence of infection or intubation during their ICU admission; and (3) to evaluate mortality in patients with intubation during their ICU stay.

### Data collection

Clinical and laboratory data, and other ICU events of patients were recorded daily. The impact of demographic characteristics, laboratory tests, underlying diseases, drug histories, and other findings on study outcomes were analyzed. Patients were followed until death or discharge from the hospital. A team of three clinicians (A.S., A.G., M.M.) was responsible for registering daily evaluations of patients in the ICU. Baseline information consisting of medical and drug histories, laboratory tests, and other details at admission was obtained from patients’ medical files. A special COVID-19 form was designed to document all the above information. The collection process was supervised by F.N.

### Treatments

The ICU patients received strict volume management, multiorgan function evaluation, nutritional assessment, and appropriate nutritional support. At the time of the study, vasopressors (such as norepinephrine), non-steroidal anti-inflammatory drugs (such as paracetamol), broad-spectrum antibiotics, corticosteroid therapy (such as hydrocortisone and corticosteroid inhalers), bronchodilators (such as ipratropium bromide and salbutamol sulfate inhalers), hydroxychloroquine, and anticoagulants (like enoxaparin) were used for these patients according to our COVID-19 national protocol in Iran^9^.

### Statistical analysis

We expressed quantitative variables as median and interquartile (for abnormally distributed variables) or mean ± SD (for normally distributed variables), and qualitative variables as numbers and percentages. We also performed the chi-square tests for categorical variables, and the Mann–Whitney U tests for quantitative variable comparisons. Using the Kaplan–Meier method, 92-day survival analyses were calculated, and the significance was assessed with the Log-Rank (Mantel-Cox) test. A multi-variate logistic regression model was created from some mortality factors to predict each unvaccinated ICU-admitted patient’s probability of death. In order to determine the discrimination of the APACHE II score, which was evaluated at ICU admission, the area under the receiver-operating characteristic curve (AUC-ROC) was calculated with a 95% CI. AUROC of greater than 0.5, greater than  0.6, greater than  0.70 or  greater than  0.80 were considered poor, fair, satisfactory or good, respectively^10–12^. Statistical significance was assumed for *P* values equal or less than 0.05, while marginal significance was stated for the range of *P* values between 0.05 and 0.10. SPSS version 22 was used for all statistical testing.

## Results

The present study included 93 unvaccinated patients diagnosed with COVID-19 and admitted to the ICU, of whom 41 survived. The mean duration from the start of symptoms to hospitalization was 6.92 ± 4.27 days, and from hospital admission to ICU admission was 2.52 ± 3.61 days. Patients’ average hospital stay was 13.23 ± 10.43 days, with an average of 8.84 ± 7.53 days in the ICU. As shown in Table 1, non-survivors had marginally longer ICU lengths of stay than survivors (*P* = 0.06). Also, non-survivors had significantly higher APACHE II scores (*P* = 0.02). (Table 2). A significantly increased number of non-survivors were intubated in comparison to survivors (*P* < 0.05). Vasopressor use during ICU admission was marginally higher in the non-survivor group (*P* = 0.06).

**Table 1:** Evaluation of COVID-19-infected patients during ICU admission

Variables	Survivors	*n*	Non-survivors	*n*	*P*
Intubation during ICU admission	11 (26.82)	41	43 (84.31)	51	0.001*
Vasopressor use during ICU admission	7 (20)	35	20 (39.21)	51	0.04
Days of stay in ICU	5 [3–11]	35	8 [5–11.75]	52	0.06
Total days of hospital stay	10 [7–18.75]	40	10.5 [5–16]	52	0.6
Complications					
Acute kidney injury	9 (37.5)	24	17 (43.58)	39	0.45
Concomitant infection during ICU admission	13 (37.14)	35	24 (48)	50	0.13
UTI	1 (2.85)	35	2 (4)	50	NA
VAP	6 (17.14)	35	14 (28)	50	
HAP	6 (17.14)	35	8 (16)	50	

Numerical data are presented as median [1st–3rd quartile] or n (%); χ^2^ test for categorical variables. Mann–Whitney U test for quantitative variables.

HAP, hospital associated pneumonia; NA, not applicable; UTI, urinary tract infection; VAP, ventilator associated pneumonia.

* *P* < 0.05 was considered as significant.

**Table 2:** Evaluations of COVID-19-infected patients documented at ICU admission

Variables	Survivors	*n*	Non-survivors	*n*	*P*
Temperature (°C)	37.5 [37–38]	35	37.6 [37–38.2]	51	0.39
Respiratory rate (breaths / min)	20 [18–22]	35	20 [18–24]	51	0.73
SpO2 at admission (%)	94 [90–97]	35	92 [87–95]	51	0.08
Patient weight (kg)	75 [70–85]	35	75 [60–80]	51	0.14
White blood cell (cells * 10^3^/ µl)	8.3 [4.45–13.33]	41	9 [0.9–14.8]	51	0.3
Lymphocyte (%)	16.6 [8.4–27]	39	12.1 [8–18.8]	50	0.17
Neutrophil (%)	76.5 [61.4–86.3]	39	79.9 [75.05–87.1]	49	0.13
Haemoglobin (g/dl)	12.20 [10.6–13.6]	41	11.35 [9.6–13.65]	52	0.05 *
Platelet count (cells * 10^3^/ µl)	201 [151.5–268]	41	192.5 [158.25–260]	52	0.91
Urea (mg/dl)	39 [22-51]	41	42 [25 – 58]	52	0.25
Creatinine (mg/dl)	1.20 [0.95 – 1.5]	41	1.15 [0.82 – 2.07]	52	0.46
Aspartate aminotransferase (u/l)	37.50 [25.25 – 56]	28	36.50 [24 – 77.5]	46	0.95
Alanine aminotransferase (u/l)	26 [15 – 38.75]	28	19.50 [11 – 43.25]	46	0.51
Alkaline phosphatase (u/l)	223 [171.75 – 271]	28	232 [183 – 315]	45	0.3
Total bilirubin (mg/dl)	0.9 [0.7 – 1.5]	26	0.9 [0.7 – 1.2]	44	0.49
C-reactive protein (mg/dl)	34.95 [15.17 – 63.52]	40	35.50 [22.8 – 92]	51	0.39
Erythrocyte sedimentation rate (mm/h)	40 [39.25 – 60]	36	43.50 [25.75 – 71.25]	50	0.66
CPK	218.50 [127.25 – 487]	34	113 [54.5 – 368]	49	**0.02** *
LDH	549 [453 – 720]	35	599.50 [477.25 – 848.25]	50	0.25
BS (mg/dl)	117 [95 – 179]	41	128 [98 – 206]	51	0.49
INR	1 [1 – 1.1]	40	1 [1 – 1.2]	50	0.38
PCT	0.55 [0.13 – 3.95]	11	0.3 [0.05 – 0.91]	14	0.42
Albumin	3.40 [3 – 3.8]	23	3.20 [2.77 – 3.7]	38	0.43
GFR (ml/min)	62.70 [45.55 – 78.5]	40	58.8 [32.42 – 79.92]	50	0.91
D-dimer	1777.29 [569-3051.96]	5	8.8 [3.79 – 8196.5]	13	0.58
Blood Groups					
A	9 (25)	36	13 (25.49)	51	0.96
B	17 (47.22)	36	14 (27.45)	51	0.06
AB	0 (0)	36	4 (7.84)	51	0.09
O	10 (27.77)	36	20 (39.21)	51	0.27
Rh					0.06
Positive	19 (52.77)	36	37 (72.54)	51	
Negative	17 (47.22)	36	14 (27.45)	51	
APACHE II score at ICU admission	12 [8–18]	35	17 [11 – 24]	51	**0.02** *
Days from symptoms onset to ICU admission	7 [5 – 9]	24	7 [3 – 10]	39	0.58
Days from hospital admission to ICU admission	1 [0 – 3]	35	2 [0 – 4]	51	0.18
Symptoms at the time of hospital admission	27 (77.14)	35	46 (90.19)	51	0.1
Nausea	2 (5.71)	35	6 (11.76)	51	0.34
Vomiting	1 (2.85)	35	6 (11.76)	51	0.14
Dyspepsia	1 (2.85)	35	1(1.96)	51	0.79
Dry cough	18 (54.42)	35	22 (43.13)	51	N/A
Fever	18 (54.42)	35	26 (50.98)	51	0.97
Chills	8 (22.85)	35	10 (19.60)	51	0.72
Dyspnoea	23 (65.71)	35	36 (70.58)	51	0.63
Fatigue	8 (22.85)	35	12 (23.52)	51	0.94
Myalgia	9 (25.71)	35	13 (25.49)	51	0.98
Headache	2 (5.71)	35	2 (3.92)	51	0.7
Diarrhoea	2 (5.71)	35	2 (3.92)	51	0.7
AMS	4 (11.42)	35	13 (25.49)	51	NA
Type of oxygen therapy first day in ICU					NA
Nasal cannula	10 (28.57)	35	8 (15.68)	51	
Reservoir bag	14 (40)	35	23 (45.09)	51	
NIV/CPAP	6 (17.14)	35	8 (15.68)	51	
Invasive MV	5 (14.28)	35	12 (23.52)	51	
Age, years	59 [42–69.5]	41	65 [53.25–77]	52	0.03*
Sex					
Male	23 (56.09)	41	26 (50)	52	0.56
Female	18 (43.90)	41	26 (50)	52	
Underlying disease	27 (77.14)	35	48 (94.11)	51	**0.02** *
Hypertension	14 (40)	35	23 (45.09)	51	0.64
Diabetes mellitus	9 (52.71)	35	21 (41.17)	51	0.14
Cardiovascular disease	8 (22.85)	35	14 (27.45)	51	0.63
Chronic kidney disease	4 (11.42)	35	8 (15.68)	51	0.58
Malignancy	2 (5.71)	35	9 (17.64)	51	0.11
Neurologic disorder (CVA, Parkinson, Alzheimer)	2 (5.71)	35	9 (17.64)	51	0.1
Chronic lung disease	2 (5.71)	35	5 (9.80)	51	0.5
Autoimmune disease	1 (2.85)	35	6 (11.76)	51	0.14
Thyroid disease	2 (5.71)	35	5 (9.80)	51	0.5
Chronic liver disease	1(2.85)	35	1(1.96)	51	0.79
Addiction	3 (8.57)	35	6 (11.76)	51	0.64
Immunosuppressive agents use	2 (5.71)	35	11 (21.56)	51	**0.04** *

Numerical data are presented as median [1^st^–3^rd^ quartile] or *n* (%) χ^2^ test for categorical variables. Mann–Whitney U test for quantitative variables.

AMS, altered mental status; APACHE II, Acute Physiologic and Chronic Health Evaluation II; BS, blood sugar; CPK, creatine phosphokinase; GFR, glomerular filtration rate; INR, international normalized ratio; LDH, lactate dehydrogenase; MV, mechanical ventilation; NA, not applicable; NIV/CPAP, noninvasive ventilation/continuous positive air pressure; PCT, procalcitonin; Rh: Rhesus; SpO2, oxygen saturation.

* *P* < 0.05 was considered as significant.

The types of clinical symptoms and oxygen therapy administered at hospital admission are listed in Table 2. COVID-19 complications development during patients’ ICU stays, such as acute kidney injury (AKI) and concomitant infections, were also evaluated (Table 1).

### Demographics and comorbidities

**Figure 1:**
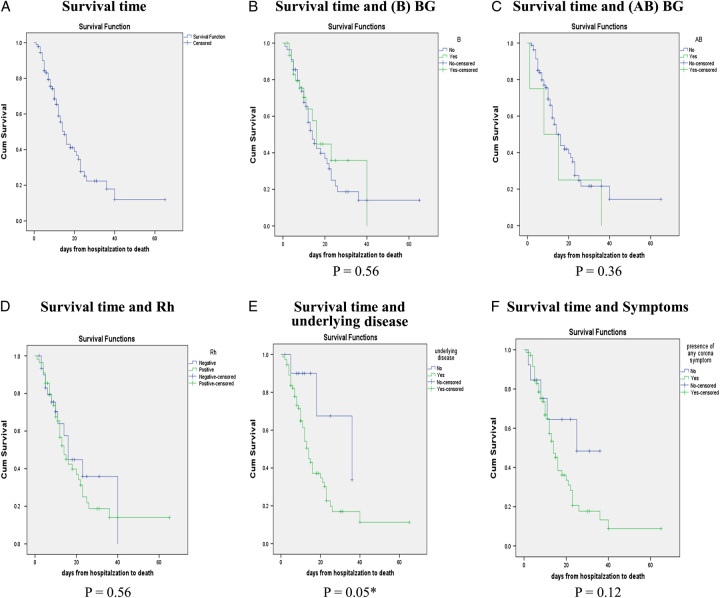
Survival time analysis. Survival time analysis was done by Kaplan–Meier and *P* values were calculated with Log-Rank (Mantel–Cox) Among factors that were analyzed, only underlying disease could significantly decrease survival time in patients. BG, blood group, Rh, Rhesus, *: significant, *P* < 0.05.

The non-survivors group was significantly older than survivors [65 (53.25–77) vs. 59 (42–69.5) years old, *P* < 0.05], and they had a considerably higher number of patients with underlying disease (48 vs. 27, *P* < 0.05). Accordingly, as illustrated in Fig. 1(E), having an underlying disease can greatly reduce patients’ survival time (Log-Rank test, *P* = 0.05). However, these two groups had no significant difference when their comorbidities were compared separately. Moreover, the use of immunosuppressive agents was significantly more prevalent among non-survivors than among survivors (11 vs. 4, *P* < 0.05).

### Laboratory measurements and vital signs

Laboratory tests were recorded for patients at hospital admission (Table 2). Patients who did not survive had significantly lower haemoglobin levels as well as higher serum levels of creatine phosphokinase (CPK) compared to survivors (*P* < 0.05). Also, non-survivors had marginally lower SpO2 than survivors (*P* = 0.08). However, some tests were not evaluated because of kit shortages during the pandemic for some patients. **Tables 1 and 2** provide specific details on the number of patients whose tests were evaluated.

### Blood group and rhesus factor

A total of 87 patients’ blood groups and Rhesus (Rh) factors were tested (six patients’ blood groups and Rh factors were not reported). As presented in Table 2, having blood group B or Rh-negative blood could be preventive against death, although this prevention was only marginally significant (*P* = 0.06). However, it is important to mention that having blood group B or a positive or negative Rh factor did not improve patients’ survival time (Fig. 1). It should be noted that blood type B or the Rh-negative phenotype did not affect days from hospitalization to death (Log-Rank test, *P* = 0.56).

### Survival time and predictor factors of mortality

Our study showed that the cumulative survival probability for patients decreased over time (Fig. 1). The mean survival was 21.77 (95% CI, 16.39–27.15) days, and the median survival was 15 (95% CI, 12.13–17.86) days.

**Figure 2:**
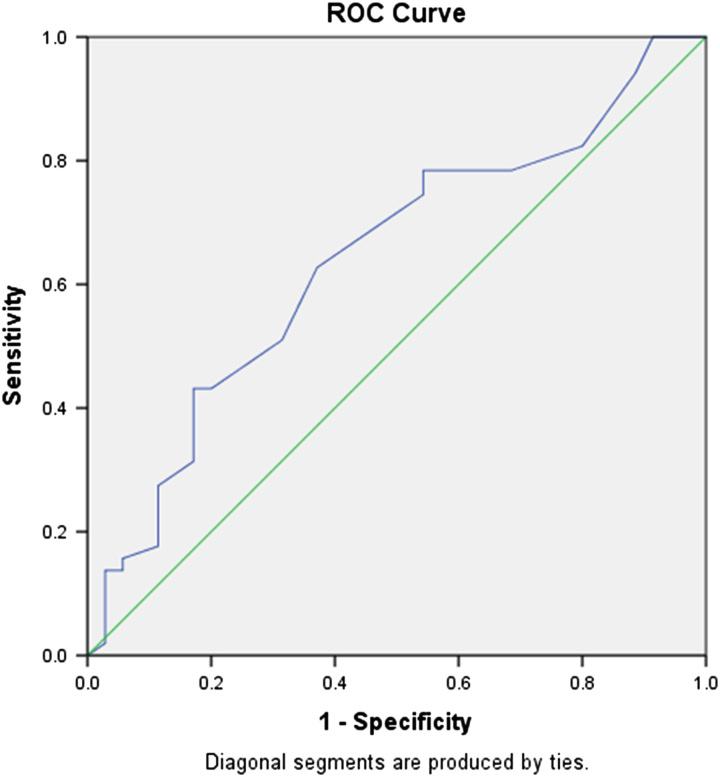
APACHE II at ICU admission receiver operator curve (ROC). The area under curve (AUC) was 0.64 (95% CI 0.52–0.75, *P* < 0.05). Cut-off point: 14.5, with sensitivity: 62.7%, and specificity: 62.86%.

A logistic regression model was constructed using the APACHE II score and underlying disease as variables related to mortality among COVID-19 patients after ICU admission. (Table 3). Accordingly, a one-point increase in APACHE II score increased the mortality rate by 6% (95% CI, 0.3–12%), and with the presence of underlying disease, patients’ mortality increased by 4.27 times (95% CI, 0.003–18.24). We used ROC curve analysis to determine whether the APACHE II score predicts prognosis for individuals admitted to the ICU with COVID-19 (Fig. 2). The AUC of APACHE II was 0.64 (95% CI, 0.52–0.75, *P* < 0.05). The cut-off value was 14.5 and sensitivity and specificity were respectively 62.7% and 62.86%.

**Table 3:** Multivariate logistic regression analysis of risk factors for mortality in intensive care unit patients with COVID-19

Variable	OR	SE	*P*	Lower CI (95%)	Upper CI (95%)
APACHE II at ICU admission	1.06	0.03	0.05	1.003	1.127
Underlying disease	4.2	0.74	0.02	1.003	18.241

APACHE II, Acute Physiologic and Chronic Health Evaluation II; OR, odds ratio; SE, standard error.

## Discussion

We analyzed unvaccinated critically ill ICU patients with COVID-19 and interpreted their demographics, clinical characteristics, comorbidities, and laboratory data upon ICU admission for a better understanding of their impact on survival. The overall mortality rate of our patients was 55.9%. The global rate of mortality was 32.6%, with Middle Eastern and North African countries having a mortality rate of 61.9% during the pandemic^4,13^. In Iran, many ICUs were overloaded beyond their capacity during the pandemic. A major concern is that Iran’s healthcare system has fewer ICU beds per capita than developed countries, that is 4.92 per 100 000 inhabitants and 7.6 per 100 000 in our province, precisely^14^. To compare this with more developed countries, Belgium has 15.9 ICU beds per 100 000 people, and Germany has 29.2, considerably higher than a lower-middle-income country like Iran^15^. Consequently, this shortage can result in a high mortality rate in our country.

According to our study, older age is a decisive factor contributing to increased mortality, and other studies support this finding^16,17^. Additionally, underlying diseases could significantly impact mortality rates and survival times. Similarly, based on other studies, underlying conditions (such as cardiovascular disease, hypertension, and diabetes mellitus) are one of the main risk factors for mortality in patients with COVID-19^18^.

A significant increase in CPK levels was observed in non-survivors. The elevation of CPK indicates muscle injury, partially explaining the increased chance of mortality in the ICU. With high CPK levels, patients are more likely to receive invasive treatments in the ICU, and their muscle strength and mass may decline even more as time passes in the ICU^19^. ESR serum levels in our ICU-admitted patients were elevated in survivors (40 mm/h) and non-survivors (43.5 mm/h). Evidence shows that ESR levels also increased in COVID-19 patients suffering from severe disease and pneumonia^20^. Among ICU-admitted patients, we found increased CRP concentration in survivors (34.95 mg/dl) and non-survivors (35.5 mg/dl). CRP is a protein produced by the liver that is an early indicator of infection and inflammation. Additionally, it helps assess the severity of COVID-19^21^.

The median LDH levels were higher than normal in ICU-admitted non-survivors (599.5 U/l) and survivors (549 U/l). During acute and severe lung injury, LDH increases as an inflammatory marker and as a general indicator of tissue damage^22,23^. The median serum D-dimer levels among 5 survivors and 13 non-survivors were 1777.29 and 8.8, respectively, higher than the normal range. Based on a study conducted during the pandemic in Wuhan, elevated levels of D-dimer, particularly in the range of 10–40 μg/ml, were associated with an increased 14-day mortality rate in ICUs and a greater tendency to develop organ dysfunction in critically ill patients with COVID-19^24^. This contradiction can be justified by the unavailability of kits and resources to assess laboratory tests for all of our patients. Among the inflammatory laboratory tests examined in our study, 11 survivors and 14 non-survivors had normal PCT values. Increasing PCT levels may be attributed to bacterial co-infections and can thus be utilized to begin antibiotic treatment^25^. Our results did not necessarily imply the absence of bacterial co-infection, as the minimal availability of PCT testing kits may have resulted in undiagnosed patients with a higher PCT count among our patients. Anaemia and low haemoglobin levels were associated with a significantly increased mortality rate in our patients. Our findings align with those of Jha M *et al*.^26^. According to their retrospective study of 784 admitted COVID-19 patients, anaemia at admission was a predictor of death due to the virus infection. Our study results showed that non-survivors had lower SpO2 levels than survivors. SpO2 values are usually used to evaluate the extent of respiratory incapacity in patients infected with COVID-19, as it usually involves the lungs^27,28^.

In our study, 54 patients (58%) were intubated during their ICU admission, and being intubated was highly significant in non-survivors compared to survivors. The morbidity and mortality rates are very high in patients with acute respiratory distress syndrome (ARDS), who progress to mechanical ventilation^29^. As compared to ARDS associated with other conditions, COVID-19 ARDS has poorer outcomes, with mortality rates ranging from 65.7 to 94% for mechanically ventilated patients^30^. Vasopressor treatment was more commonly administered to non-survivor patients than to survivors. Many patients require vasopressors to manage hypotension arising from sepsis due to primary virus infection or concomitant bacterial infection^31,32^.

Our findings suggest that the APACHE II score can be useful in predicting mortality in critically ill patients infected with COVID-19. Cheng *et al*.^33^. also demonstrated that the APACHE II score was a more accurate predictor of disease severity and mortality in COVID-19 patients than CURB-65 (confusion, urea, respiratory rate, blood pressure, and age ≥ 65) and MuLBSTA (multilobular infiltration, hypo-lymphocytosis, bacterial co-infection, history of smoking, hypertension, and age ≥ 65). On the contrary, in a study conducted by Isted *et al*.^34^. , non-survivors and survivors did not have different APACHE II scores. Multi-variate logistic regression analysis demonstrated that baseline APACHE II score and preexisting underlying disease were significant mortality risk factors in COVID-19 unvaccinated patients.

These findings highlight the importance of early identification and management of risk factors in COVID-19 ICU-admitted patients, especially when unvaccinated. The global spread of COVID-19 has caused healthcare systems to grapple with never-before-seen difficulties, especially in forecasting morbidity and mortality in these patients. The lack of knowledge about the disease until the pandemic delayed the coordination of guidelines and treatment plans. As a result, healthcare professionals and researchers had to work quickly to develop effective treatments and protocols in response to the outbreak. Thus, having a reliable clinical predictor for mortality is beneficial for making optimal decisions for the future^7^.

Our study had some limitations worth noting. One common limitation in longitudinal studies, including ours, is missing data. This was due to healthcare providers and products limitations. Additionally, being a single-centre study may restrict the ability to generalize our findings to other populations. Furthermore, the small patient population is another limitation of this study. Due to the study’s observational nature, we did not have a control group. Finally, we were unable to consider the impact of different treatment modalities on mortality in our study.

This analysis, needs multi-centre, large-scale studies to attain more generalizable results. Further research can find other potential mortality predictors in ICU-admitted COVID-19 patients. Future studies could also investigate the impact of different treatments and interventions on mortality rates in COVID-19 patients admitted to the ICU to help guide clinical decision-making and improve patient outcomes.

## Ethical approval

The research was approved by the Mazandaran University of Medical Sciences ethics committee with the approval code IR.MAZUMS.REC.1399.351.

## Consent

Patients’ informed written consent (or from legally authorized substitute decision-makers) were obtained in Medical and Educational Imam Khomeini Hospital. The PDF format of the consent is available from the corresponding author, upon reasonable request.

## Sources of funding

This study has not been funded by any individuals or organizations.

## Author contribution

F.H. and E.K.R.: study design; coordination of the research; supervising data collection. P.A.: writing and editing the manuscript; critically reviewing the manuscript for important intellectual content; designing the graphical abstract. A.V.: writing and editing the manuscript; data analysis and interpretation. A.S., A.G.B., and M.M.: designing the data collection instruments, data collection, and registration. A.A.: data analysis and interpretation. M.M.: data analysis and critical review. F.N.-B.: writing the initial manuscript; supervising data collection; reviewing and revising the manuscript.

## Conflicts of interest disclosure

There is no conflicts of interest with this study.

## Research registration unique identifying number (UIN)

Unfortunately, due to severe sanctions imposed on our country, Iran, international credit card transactions are not feasible from our location. Therefore, we could not register for a UIN on the relevant websites. We were unable to use foreign credit cards because only individuals from outside the country could make the payments. Free UIN registration sites were also inaccessible to us because they only accepted registrations from their own countries.

## Guarantor

Fahimeh Naderi-Behdani.

## Data availability statement

The data supporting the findings of this study are available within the article. Raw data that support the findings of this study are available from the corresponding author, upon reasonable request.

## Provenance and peer review

Not commissioned, externally peer-reviewed.
